# Biochar-Induced Changes in Soil Hydraulic Conductivity and Dissolved Nutrient Fluxes Constrained by Laboratory Experiments

**DOI:** 10.1371/journal.pone.0108340

**Published:** 2014-09-24

**Authors:** Rebecca T. Barnes, Morgan E. Gallagher, Caroline A. Masiello, Zuolin Liu, Brandon Dugan

**Affiliations:** Department of Earth Science, Rice University, Houston, Texas, United States of America; Purdue University, United States of America

## Abstract

The addition of charcoal (or biochar) to soil has significant carbon sequestration and agronomic potential, making it important to determine how this potentially large anthropogenic carbon influx will alter ecosystem functions. We used column experiments to quantify how hydrologic and nutrient-retention characteristics of three soil materials differed with biochar amendment. We compared three homogeneous soil materials (sand, organic-rich topsoil, and clay-rich Hapludert) to provide a basic understanding of biochar-soil-water interactions. On average, biochar amendment decreased saturated hydraulic conductivity (*K*) by 92% in sand and 67% in organic soil, but increased *K* by 328% in clay-rich soil. The change in *K* for sand was not predicted by the accompanying physical changes to the soil mixture; the sand-biochar mixture was less dense and more porous than sand without biochar. We propose two hydrologic pathways that are potential drivers for this behavior: one through the interstitial biochar-sand space and a second through pores within the biochar grains themselves. This second pathway adds to the porosity of the soil mixture; however, it likely does not add to the effective soil *K* due to its tortuosity and smaller pore size. Therefore, the addition of biochar can increase or decrease soil drainage, and suggests that any potential improvement of water delivery to plants is dependent on soil type, biochar amendment rate, and biochar properties. Changes in dissolved carbon (C) and nitrogen (N) fluxes also differed; with biochar increasing the C flux from organic-poor sand, decreasing it from organic-rich soils, and retaining small amounts of soil-derived N. The aromaticity of C lost from sand and clay increased, suggesting lost C was biochar-derived; though the loss accounts for only 0.05% of added biochar-C. Thus, the direction and magnitude of hydraulic, C, and N changes associated with biochar amendments are soil type (composition and particle size) dependent.

## Introduction

Woolf et al. [1] estimate that 1.8 Pg CO_2_-carbon equivalents can be sequestered each year through the sustainable production and application of 0.9 Pg of biochar to agricultural land which sequesters carbon (C), reduces CH_4_ and N_2_O emissions, and results in avoided CO_2_ emissions. Biochar, charcoal intentionally produced by humans through pyrolysis for soil amendment, is a type of black carbon, like soot or charcoal [2], [3]. Adding 0.9 Pg of biochar to the agricultural landscape would correspond to a 4–20 fold increase in global black carbon production (0.04 to 0.194 Pg yr−1; [4]). Recent work illustrates the likely mobility of biochar added to soil: charcoal in soils can be released into rivers [5], [6], where, given its aromatic structure, it can be photo-oxidized [7] and/or transported downstream where it has the potential to alter ecosystem processes [6]. Given the potential magnitude of ecosystem perturbations from full-scale implementation of biochar-C sequestration, it is critical to determine the effects of biochar soil amendment on water and biogeochemical cycling.

Biochar soil amendment can increase crop productivity [3], [8], potentially by improving the hydrologic properties of the soils [9]. Biochar can also increase soil water-holding capacity, and available water content [8], [10]–[13], plant available water [14]–[16], alter soil hydrophobicity [17], and change soil hydraulic conductivity [18]–[22]. Biochar is predicted to cause sandy soils to drain more slowly [23] and clay-rich soils to drain more rapidly [24]. However, past results have not been consistent, likely due to confounding factors such as biochar characteristics (i.e. feedstock and pyrolysis temperatures), application rates, and soil characteristics. Given the importance of hydraulic conductivity in determining the partitioning of precipitation between infiltration and overland flow (i.e. infiltration rates; [25]), which impacts water storage in the subsurface and thus plant available water, it is necessary to understand the effects of biochar on the hydraulic properties of different soil types.

The C and nitrogen (N) content of biochar varies with feedstock and production conditions [26]. These conditions and the C∶N ratio of biochar influence its stability [27], [28] as well as possible soil C and N losses [29]. While dependent on production conditions, biochar tends to have a high cation exchange capacity [30] and anion sorption ability [31], allowing for adsorption of dissolved organic matter (DOM) [32] and N [33], and can alter greenhouse gas emissions [34]–[36]. While biochar amendment adds C and N to soils (which may be available for leaching), it is also able to sequester additional C and nutrients in the soil due to its sorptive properties. Therefore the addition of biochar to soil could result in a net increase or decrease in dissolved C and N losses [37]. Worldwide analysis of dissolved black carbon (DBC), which includes derivatives of charcoal and biochar, exported from rivers indicates that, on average, DBC contributes 10% of the global total dissolved organic carbon (DOC) flux [6]. In addition, analysis of the bioavailability of biochar extracts in natural stream water suggests that some biochar-C molecules have turnover rates on the order of days to a month [38], indicating that at least a portion of biochar-C is not recalcitrant. In addition to the dissolution of biochar within soils, particles are also transported. Recent studies illustrate that the movement of biochar particles is related to particle size and surface chemistry, as well as pore water salt content and pH [39], [40]. Collectively this research points to the uncertainty in the fate of biochar and biochar-C and their down-gradient effects on aquatic ecosystems.

Using column experiments, we quantify the effects of a 10% (by mass) biochar amendment on the saturated hydraulic conductivity (*K*) of sand, clay-rich, and organic-rich soil materials as well as report the effects of this soil amendment on C and N leaching. Using simple, homogeneous soil materials with different grain sizes and surface chemistry allowed us to examine biochar-soil interactions and to compare our results to established soil hydrology models. These data begin to address an important knowledge gap by providing new quantitative constraints on how biochar amendments change *K* and the chemistry of soil leachate; this work points to the need for more mechanistic studies to examine biochar-soil-water interactions.

## Materials and Methods

### Soil and Biochar

Three soil materials were used to gauge soil property responses to biochar amendment. Sand (Pavestone Natural Play Sand) and organic-rich soil (Micro-Gro Organic Rich Garden Soil, with no N fertilizer) were purchased from Home Depot. The organic-rich soil was texturally similar to a sandy loam. A clay-loam Hapludert, characterized by its poor drainage [41], was collected from Rice University's campus. All materials were oven dried at 60°C to remove any moisture prior to dry sieving. Dry materials were mixed, and then oven dried at 100°C for 24 hours to create homogenous mixtures with initial water content of zero; 100°C facilitates water loss but minimizes chemical impacts as it is significantly lower than our pyrolysis temperature [42]. We determined the grain size of the three soils and the biochar using seven sieves (38 µm to 500 µm).

We produced biochar from mesquite wood (*Prosopis* sp.), ground to smaller than 20 mesh (850 µm). Batches of mesquite (70–80 g) were pyrolyzed using the reactor design described in Kinney et al. [17], by heating in a muffle furnace at 6°C min^−1^ and holding at 400°C for 4 hours. On average these pyrolysis conditions provided a biochar mass yield of 40.4%. The produced biochar had a pH of 6.5±0.1, ash content of 3.33%±0.04%, and liming equivalent of 4%, determined using protocols outlined by the International Biochar Initiative [43]–[45].

### Column Experiments

To test the response of *K* and dissolved nutrient fluxes to biochar amendment we conducted falling head experiments [46] across six materials: sand, sand+biochar, organic soil, organic soil+biochar, clay, and clay+biochar. We packed 150 g of each mixture into three replicate columns, 50 g per column, with 54 µm polyester mesh screen (Small Parts Inc.) at the bottom. Materials were packed with a consistent force into columns in four equal increments to achieve uniform bulk density [47] and the initial soil length was recorded. Bulk density for soil materials and soil+biochar mixtures was determined using the dry mass and column dimensions (height of soil materials, diameter of column) at the start of the experiment. Grain size distributions of soil+biochar mixtures were estimated using the proportional masses of each material (i.e. 10% biochar, 90% soil material) and appropriate grain size data (Table 1).

**Table 1:** Physical and elemental properties of soil materials and biochar.

Material	Grain Size			Bulk Density	%C	%N
	d_10_ (µm)	d_50_ (µm)	d_90_ (µm)	ρ_d_ (g cm^−3^)		
sand	70	160	380	1.68±0.18	0.4	0.01
organic-rich	95	400	480	0.43±0.002	37.9	0.54
clay-rich	45	115	460	1.72±0.04	0.9	0.03
biochar (mesquite)	75	320	470	0.36±0.03	71.6	0.84

Biochar constituted 10% of the total mass in the columns that contained biochar. This represents a 133 tons ha^−1^ (95 Mg C ha^−1^) application rate with a 10 cm tillage depth. We chose a high amendment rate to ensure that we altered the soil-water system in a way that would allow us to detect any effects across the three contrasting soil materials. Specifically, we were concerned about the potential of soil biochar amendments increasing C exports to surface waters, as well as any unforeseen consequences on soil hydrologic properties. This amendment rate is above what is likely to be added to an agricultural field, though within the range reported for positive or neutral productivity effects (up to 140 Mg C ha−1; [3]), similar to the application made by Chan et al. [48], and well below what was shown to be an upper limit to biochar-induced benefits to plants (200 tons C ha−1; [15]).

To saturate the soils, we capped the column bottoms, added 150 mL of 18 MΩ-cm MilliQ water, and allowed the columns to sit for 48 h before drainage. Six consecutive falling head experiments [i.e. flushing events; [46] were conducted on saturated soils using 150 mL of MilliQ water, with leachate collected at the end of each experiment for all columns. The same experimental set up was used for all six materials to allow for inter-comparison. The leachate was weighed, filtered through a pre-combusted glass fiber filter (Whatman GF/F) and kept at 4°C until analysis. This process was repeated without allowing columns to dry between flushing events. Evaporation was monitored daily (via the net change in the mass of water in a beaker) and water throughput (leachate volume) was corrected for evaporation by adding the product of the daily evaporative loss (mg hr^−1^) and duration of flushing event (hrs).

Saturated hydraulic conductivity (*K*) from falling head data was calculated using equation 1 [49]:
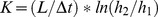
(1)where *L* is soil sample length (m), *Δt* is time elapsed (s), and *h_1_* and *h_2_* are the initial and final water heights (m), respectively (data available in Table S1). Separate experiments on sand-only systems confirm consistent measures of *K* from top-saturated and bottom-saturated falling head and constant head experiments, suggesting full saturation and steady-state conditions were achieved using this falling-head technique.

### Soil and sample analysis

We used a Shimadzu TOC-VCN to determine the DOC and total dissolved nitrogen (TDN) concentrations of the filtered leachate. Sample replicates indicate a 0.08 mg L^−1^ and 0.04 mg L^−1^ precision for DOC and TDN, respectively. Mass loss of C and N (dissolved flux) were determined by multiplying the DOC and TDN concentrations by the water volume of the sample, respectively. In contrast, water throughput was calculated using the evaporation-corrected water volumes. The aromaticity of the dissolved C was determined by calculating the specific UV absorbance at 254 nm (SUVA254; [50]). The UV absorption (m^−1^) of filtered leachate was measured on a Cary UV-Vis spectrophotometer and divided by the DOC concentration (mg C L^−1^) following the protocol developed by Weishaar et al. [50] to calculate SUVA_254_ (L mg C^−1^ m^−1^).

After six flushing events, soil mixtures were removed from the columns, weighed, dried at 100°C for at least 24 hours, and reweighed to determine water content at field capacity by mass. We measured C and N content on original soils, biochar, and dried, post-experiment soils using a Costech 4010 CHNS/O Elemental Analyzer. Replicate analysis shows a precision of 0.6% and 0.02% for C and N measurements, respectively.

### Statistical analyses

We used two-sample t-tests to determine statistical differences in the soil and leachate characteristics between treatments and controls. Paired t-tests were used to determine the statistical differences of *K* over the course of the experiment. Finally, a general linear model with flush number as a covariate, was used to determine if *K* changed significantly over the course of the experiment as a result of the biochar amendment. All statistical tests were done in the RStudio environment (v0.98.507, 2014 RStudio, Inc.) and results were considered significant when *p*<0.05.

## Results and Discussion

### Soil physical characteristics

The addition of biochar to the soil materials changed a number of physical properties, e.g. grain size distribution and bulk density, which likely affected water movement. The grain size distribution of biochar differed from that of the three soils and was most similar to the organic soil (Table 1). When biochar was added to sand and clay soils, the d_50_ of the mixtures increased; however, when biochar was added to the organic soil the d_50_ decreased. Given the similarity in d_10_ of sand and biochar, the addition of biochar did not appreciably change the proportion of fines in the sand versus the sand+biochar mixture. The addition of 10% biochar changed soil bulk density (ρ_b_) (Table 2), though these changes were not always significant; the addition of biochar decreased the ρ_b_ of sand and clay by 17% (p = 0.056) and 20% (p = 0.052), respectively. Biochar addition to clay lowered ρ_b_ enough to bring it within the range recommended by the National Soil Conservation Service to allow for adequate root growth (<1.47 g cm^−3^, USDA, 2008). In contrast, when biochar was added to organic soil the mixture ρ_b_ increased 10% (p = 0.018), despite the biochar having a lower ρ_b_ (organic: 0.43±0.002 g cm^−3^; biochar: 0.36±0.03 g cm^−3^; Table 1); this is likely related to the smaller relative grain size of biochar and grain arrangement during packing of the columns (Table 1).

**Table 2:** The mean (and standard deviation) of physical, hydraulic, and nutrient properties of the three replicates of each soil and soil+biochar treatment.

Soil	Hydraulic conductivity (*K*) (m s^−1^)		Bulk Density (*ρ_d_*) (g cm^−3^)		Water Content at Field Capacity (fraction water)		Cumulative DOC loss (mg)		Cumulative TDN loss (mg)		SUVA_254_ (L mg C^−1^ m^−1^)	
	Soil	+biochar	Soil	+biochar	Soil	+biochar	Soil	+biochar	Soil	+biochar	Soil	+biochar
sand	2.9×10^−6^ (6.3×10^−7^)	2.3×10^−7^ (5.9×10^−8^)	1.69 (0.18)	1.39 (0.06)	0.15 (0.02)	0.30 (0.03)	1.71 (0.08)	3.29 (0.35)	0.25 (0.04)	0.19 (0.01)	1.86 (0.83)	2.75 (0.41)
*p-value*	*<0.001*		*0.056*		*0.002*		*0.017*		*0.081*		*0.043*	
organic-Rich	2.1×10^−6^ (1.9×10^−6^)	7.8×10^−7^ (7.0×10^−7^)	0.43 (0.002)	0.47 (0.008)	0.65 (0.01)	0.66 (0.02)	105.45 (3.74)	95.6 (3.07)	5.52 (0.26)	5.99 (1.25)	3.48 (0.96)	3.38 (0.73)
*p-value*	*0.038*		*0.018*		*0.757*		*0.039*		*0.565*		*0.442*	
clay-rich	3.2×10^−8^ (1.9×10^−8^)	1.2×10^−7^ (1.2×10^−8^)	1.72 (0.04)	1.38 (0.14)	0.27 (0.01)	0.33 (0.004)	4.86 (2.22)	4.03 (0.13)	0.66 (0.30)	0.25 (0.01)	2.54 (0.70)	3.75 (0.74)
*p-value*	*<0.001*		*0.052*		*0.003*		*0.587*		*0.078*		*<0.001*	

Two-tailed t-tests were conducted to determine statistical differences between control and +biochar treatments; p-values are shown in italics below mean and standard deviation values for each treatment.

### Soil hydraulic characteristics

**Figure 1:**
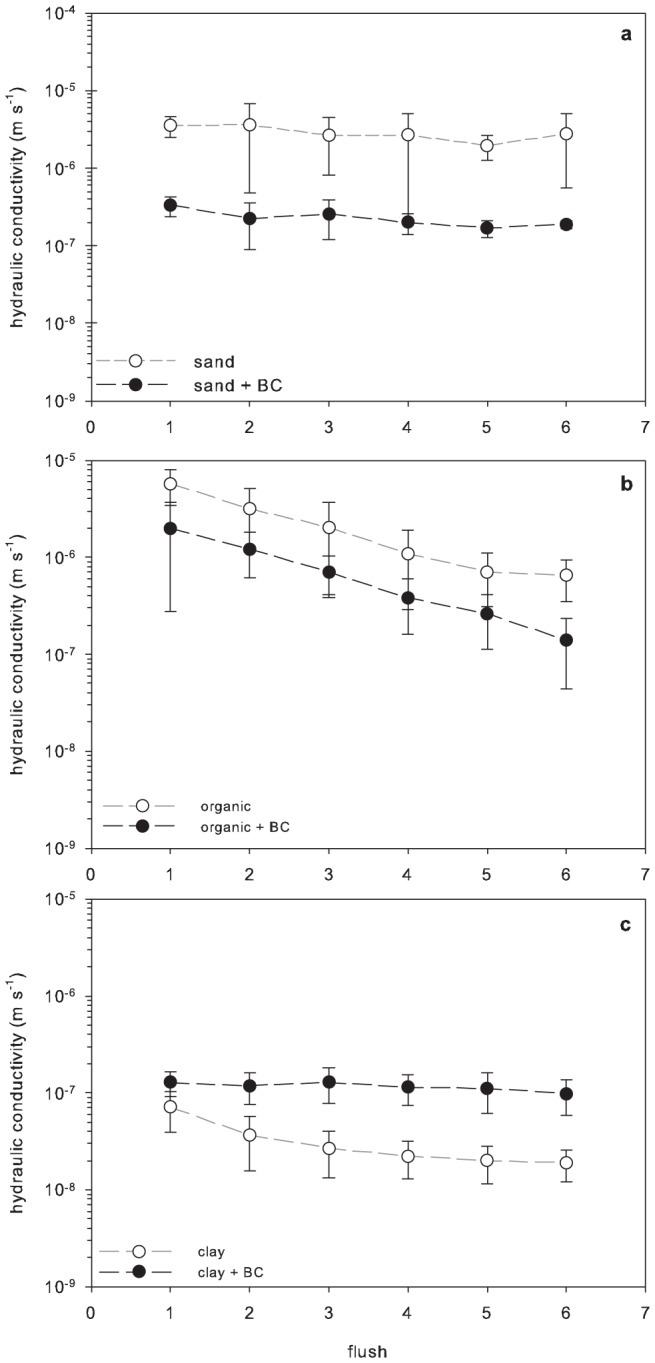
Impact of biochar amendment on saturated soil hydraulic conductivity. The saturated hydraulic conductivity (*K*), as measured using falling head experiments, for six soil treatments over subsequent flushing events: (a) sand and sand+biochar, (b) organic and organic+biochar, and (c) clay and clay+biochar. Note: the different soil treatment flushing events varied in duration with the clay (c) taking up to 10× longer to drain than the sand (a) or organic soil (b). Saturated hydraulic conductivity data and flushing duration for each flushing experiment available in Table S1.

Saturated hydraulic conductivity (*K*) describes the ease of fluid flow through saturated porous media and it can be directly measured with flow through experiments [46], [51] or estimated using theoretical or empirical models. Theoretical models, such as the Kozeny equation [52], [53] require significant knowledge of porosity, tortuosity, pore shape, grain density, and specific surface area of solid grains. Because of the difficulty constraining all these parameters, others have developed empirical models that relate porosity and *K* [54] or grain size and *K* [55]. Here we compare our *K* results with these empirical relationships using our bulk density, grain size, and porosity data. Changes in *K* accompanying biochar amendment for both clay- and organic-rich soils follow the empirical relationships discussed above; specifically the change in *K* is inversely related to the change in bulk density (or positively related to the change in porosity) caused by the biochar amendment. The addition of biochar to clay-rich soil resulted in a ρ_b_ decrease of 20% (a porosity increase), an estimated d_50_ increase of 18%, and an increase in *K* of over 300% (3.26×10^−8^ m s^−1^ to 1.16×10^−7^ m s^−1^; Figure 1c, Table 2). In contrast, when biochar was added to organic-rich soil, the mixture was 10% denser (lower porosity) with a 2% smaller d_50_, and *K* decreased by 67% (2.23×10^−6^ m s^−1^ to 7.79×10^−7^ m s^−1^; Figure 1b, Table 2). Similarly, experiments using silt loams reported an increase in *K* with biochar amendment, though there were not always corresponding decreases in ρ_b_ (Table 3, [20]). Previous work in organic-rich soils has not documented changes in *K* in response to biochar amendment (0.5 to 2% by mass) (Table 3; [56]). Differences in results are attributable to different amendment rates, biochar grain size, soil properties, and/or threshold effects of amendment rate or grain size.

**Table 3:** A comparison of studies that examined the impact of biochar amendments on soil saturated hydraulic conductivity (*K*) and bulk density (ρ_d_).

Feedstock	Temperature (°C)	Application rate^a^	Application rate (tons biochar ha^−1^)^b^	Experiment duration (d)	Soil type and/or % sand/silt/clay	Response of soil bulk density to biochar addition	Response of *K* to biochar addition	Reference
mixed hardwood lump charcoal (e.g. oak & hickory)	NR	5, 10, 20 g kg^−1^	5.5, 11, 22 t ha^−1^	500	Mesic Typic Hapludolls	decreased	no effect detected	Laird et al. 2010
*mesquite*	*400*	*10% by dry weight mass*	*133 t ha^−1^*	*1–20*	*organic rich*	*increased*	*decreased*	*this study*
commercial biochar, wood residue (e.g. teak and rosewood)	NR	4, 8, 16 t ha^−1^	4, 8, 16 t ha^−1^	60	18/34/48	NR	increased	Asai et al. 2009
					27/45/28	NR	no significant change	
corn stover	350 & 550	7.18 t C ha^−1^	350°C = 11.3 t ha^−1^	295	Alfisol (silt loam)	decreased	increased with both biochars	Herath et al. 2013
			550°C = 10.0 t ha^−1^		Andisol (silt loam)	no change	increased with 350°C, no change for 550°C	
*mesquite*	*400*	*10% by dry weight mass*	*133 t ha^−1^*	*3–23*	*clay-loam Hapludert*	*decreased*	*increased*	*this study*
dairy manure	300, 500, 700	5% by dry weight mass	61 t ha^−1^	180	loamy, 40/35/25	decreased	increased, greater increases for biochars at higher temperatures	Lei & Zhang 2013
woodchip						decreased	increased, greater increases for biochars at higher temperatures	
charcoal produced in kilns	∼300–500	ambient levels - beneath charcoal kilns	NA	NA	Haplic Acrisols	no significant change	increased	Oguntude et al. 2008
black locust	300, 400, 500	10, 20 Mg ha^−1^	10, 20 t ha^−1^	27	sandy	no change	decreased, greatest decrease seen for 500°C biochar & higher application rate	Uzoma et al. 2011
powdered wood charcoal	NR	0.5%, 1.5%, 2.5%, 5% by dry weight mass	6.3, 18.8, 31.3, 62.5 t ha^−1^	60	sandy loam	decreased	decreased with increasing biochar concentration	Devereaux et al. 2012
acacia green waste	NR	47 Mg ha^−1^	47 t ha^−1^	900	Planosol, 72.8/16.8/10.4	decreased	increased near saturated K, no effect on unsaturated K	Hardie et al. 2014
*mesquite*	*400*	*10% by dry weight mass*	*133 t ha^−1^*	*1–4*	*sand*	*decreased*	*decreased*	*this study*

a Biochar application rate reported in paper.

b Biochar application rate converted using bulk density of soils or column materials provided in the paper and assuming tillage depth of 10 cm.

NR: not reported.

NA: not applicable.

Biochar amendment rates are provided two ways: the units provided by the study and in tons biochar ha^−1^. The conversion assumed a tillage depth of 10 cm and the bulk density of the soil or column materials provided in the paper. Studies are organized by soil type, top to bottom: organic-rich soils, clay- and silt-rich soils, and sandy soils. Results from this study are in italics.

Several studies have documented threshold effects on porosity and permeability when fine-grained particles are added to a soil. Boadu [57] found that porosity decreased with increasing fines up to a threshold of ∼5% (by mass) at which point fine grain additions increased the porosity. Similarly, Crawford et al. [58] noted that porosity is lowest when the fine particle volume equals the pore space of the coarse grains; however, if fine grains are removed or added, the porosity increases.

Models relating *K* to porosity and grain size suggest that amending sand with biochar should increase *K* given the greater porosity, overall grain size, and decreased ρ_b_. A review of research reveals that this inverse relationship between ρ_b_ and *K* is preserved in most cases (Table 3). However, in our experiments biochar amendment to sand decreased the ρ_b_ and decreased *K* by 92% (2.88×10^−6^ m s^−1^ to 2.28×10^−7^ m s^−1^; Figure 1a, Table 2). Similar changes in *K* and ρ_b_ were reported by Deveraux et al. ([22]; Table 3), though this may be due to a decrease in porosity attributed to the relatively smaller d_50_ of the biochar. In our experiments, the median grain size of biochar was larger than the sand and therefore the decrease in ρ_b_ was accompanied by an increase in porosity.

In addition to grain size, how the biochar is mixed into the soils is also important. The incorporation of charcoal into sandy, Haplic Acrisols underlying kilns, resulted in a non-significant change (9%) in ρ_b_ and an 88% increase in *K* ([18]; Table 3). It is likely that the location of the charcoal within the soil column (only in the surface layer), the soil-charcoal layering, and the charcoal properties, significantly affected infiltration rates and other hydrologic properties. Furthermore, in cases where biochar amendment results in bulk density decreases and/or porosity increases, concomitant with a decrease in *K* (e.g. our results and [21]; (Table 3), changes in physical soil properties are not sufficient to explain the observed hydrologic changes.

The observed decreases in *K* despite the increased porosity and decreased ρ_b_ are likely due to the internal structure of biochar. The biochar had an average pore volume of 1.18 cm^3^ g^−1^, porosity of 0.62, and surface area of 6×10^5^ cm^2^ g^−1^ [59]. Thus the biochar has a greater porosity (typical sand has a porosity between 0.17 and 0.33 [60]) and surface area than our sand (based on d_50_, sand = 140 cm^2^ g^−1^). The highly porous structure of biochar [59], [61] creates two theoretical flow pathways, one in the interstitial space within the biochar-sand matrix and a second connecting the pores within the biochar. According to measurements made by Brewer et al. [59] the biochar we used is dominated (99%) by macropores (0.05 to 1000 µm), and therefore includes many pores larger than the diameter of a water molecule (0.28 nm). However, this second pathway likely has greater tortuosity and smaller median pore throat size due to the size of the smallest pores as well as their lack of complete connectivity [59]. While these pores contribute to the bulk density and total porosity of the mixture, they may not contribute to the effective porosity. In addition, the biochar grains likely create torturous interstitial space between the sand and biochar grains, further decreasing K. This mechanism assumes that the internal pores and surface of biochar are not hydrophobic. While we did not make hydrophobicity measurements on the mesquite biochar, multiple studies have reported that biochar amendments do not result in greater soil hydrophobicity [17], [20]. Furthermore, Briggs et al. [12] found that laboratory-produced charcoals leached with distilled water and naturally-produced charcoal collected beneath leaf litter were less water repellent than non-leached and surficial charcoal, respectively; suggesting that the hydrophobic surface compounds may be easily removed.

A second mechanism driving decreases in *K* could be related to the high field capacity of biochar [8]; i.e. water may have continued to sorb to biochar particles, contributing to the apparent decrease in *K* for some of the soil mixtures (Figure 1a). While partial saturation of column materials is possible, the measured *K* of sand-only systems from constant-head and falling-head experiments were similar, suggesting full saturation and equilibrium conditions for both experiments. Saturated hydraulic conductivity changed with repeated flushing events in four of the six soil treatments (*p*<0.01) (Figure 1). The change in *K* with flushing events was only dependent on the presence of biochar in the case of the organic-rich soil (F = 7.366, *p* = 0.01), i.e. while *K* changed over time in the unamended organic soil, the change was greater in organic+biochar columns (Figure 1b). These shifts in *K* could be related to physical mechanisms, such as swelling and grain segregation, leading to the clogging of pores, decrease in pore radii, and possibly a variation in bulk density and sample heterogeneity over the course of the experiment. Comparing soil column heights from before and after flushing events revealed that all materials swelled between 6% (sand) and 21% (organic+biochar) suggesting that bulk density changed over the course of the experiments; but biochar addition did not result in statistically different amounts of swelling. Visual observations indicate that biochar particles moved upwards within the columns during the experiment, consistent with observations made by Wang et al. [62]; however, these changes were not quantified and require further study to determine how this movement is related to flow in soil systems and how it impacts hydraulic properties.

The addition of biochar significantly (p<0.01) increased the water content at field capacity of the sand and clay. On average biochar amendment doubled the water content at field capacity in the sand and increased it by 20% in clay (Table 2). Several studies have reported increased water content in biochar-amended soils: e.g. organic-rich Mollisols [56], Amazonian *terra preta* [8], sandy loams [13], [16], [21], [22], and silt loams [20]. The increased water holding capacity is likely the result of the internal porosity of the biochar and grain-to-grain interactions; interactions that are highly dependent on soil type and biochar production conditions and are likely the reason that other studies point to mixed results [63]. The organic soil's water content at field capacity did not change with biochar addition, likely due to the already high water holding capacity of the organic soil (Table 2).

### Soil carbon and nitrogen dynamics

The addition of biochar significantly (*p*<0.05) increased the %C, by mass in all soil materials: sand increased from 0.40±0.35 to 6.98±0.5%C, clay-rich from 0.90±0.06 to 8.33±0.21%C, and organic-rich from 37.85±3.25 to 42.47±0.30%C. Similar patterns were seen for the N content of the soil materials, though not all changes were significant: %N, by mass, increased from 0.01±0.0 to 0.06±0.03%N for sand, 0.03±0.0 to 0.11±0.02%N for clay, and 0.51±0.01 to 0.54±0.06%N for organic-rich soil.

While biochar addition increased the amount of C in all soils, it did not have a universal effect on the C loss as soil leachate (i.e. DOC flux), suggesting that the release of DOC from biochar-amended soils varies with soil type. When added to the C-poor sand, biochar amendments significantly increased the aromaticity and cumulative loss of DOC (Table 2). The increase in aromaticity of the leached DOC from the biochar treatments suggests that biochar-derived C, and not native C, was lost. DOC fluxes decreased with each subsequent flushing event, suggesting that the easily leachable biochar-C was quickly depleted.

In contrast to sand, the addition of biochar to the organic- and clay-rich soils did not increase the cumulative DOC loss (Table 2). When added to C-rich organic soil, biochar significantly decreased the cumulative DOC loss and did not change the aromaticity (Table 2). This suggests that while the biochar added leachable C to the soil (as observed in the sand+biochar treatment); it is also capable of sorbing soil C. While there was no significant change in the magnitude of DOC lost when biochar was added to clay-rich soil, the aromaticity of the leachate was significantly greater when biochar was present, suggesting that while biochar-derived leachable DOC was lost, additional soil-derived C was retained within the soil-biochar matrix.

The partitioning of biochar-C between soil sequestration and leachate is partially dependent on soil type. Approximately 0.05% of the added biochar-C was lost from the sand+biochar mixture, an amount on the low end of the cumulative losses (0.1 to 1.7% of added C) reported by Mukherjee and Zimmerman [37] for similar short-term laboratory experiments. Furthermore, the SUVA_254_ measurements indicate that while biochar-C is preferentially lost with water flow-through in sandy soils, there was also a net sorption of soil-DOC in both the clay and organic-rich soils. Thus the risk of a net increase of DOC export associated with biochar soil amendments may be minimal in soils with moderate amounts of clay, silt or organic material, a conclusion that mirrors field trials and modeling results [24], [64], [65].

Given the potential use of biochar to improve soil fertility in arid, drought-prone environments with C-poor sandy soils (e.g. [21] and references therein) it is important to quantify the partitioning of C between soil and leachate in sandy soils. In particular, while our results suggest that the majority of biochar-C remains in the sandy soil, other studies have shown that this partitioning is dependent on pore water and biochar characteristics [37], [66]. For example, Bruun and others [66] found that there is a tradeoff between increased water holding capacity and increased biochar-C leachate in sandy soils; the amount of C lost was dependent on production conditions, with greater losses seen when fast- versus slow-pyrolysis biochar was added to soils. Given the results from these experiments and recent analyses of DBC in rivers [5], [6] biochar amendments could change the chemical composition of DOC exported to downstream aquatic systems. The ecosystem impacts of this change in the chemical composition of the DOC pool remain unclear [6] and require further study.

Many studies have shown that biochar is able to retain inorganic N, reducing the nitrate flux from soils [29], [33], [67]–[69], and biochar-amended sand and clay retained more N in our experiments, resulting in a 24% (p = 0.081) and 62% (p = 0.078) reduction in N losses (Table 2); though these differences were not statistically significant. The well documented sorptive properties of biochar are attributed to surficial carboxylic and other acid functional groups that provide the cation and anion exchange capacity of biochar [32], [70], [71] and are dependent on production temperatures and feedstock [30]. The lack of statistical difference with biochar amendment in our study is likely due to the relatively low N content of the soil materials and biochar (<0.8%, Table 1), the only sources of N within our columns. Thus while the results of our experiments are in line with past research, it is important to note that they do not represent the full potential of biochar to mitigate N leaching from agricultural fields.

## Conclusions

Biochar-associated changes in *K* and field capacity have implications for infiltration rates and plant water availability. As shown by our experiments, the addition of biochar to coarser soils decreases *K*, indicating the potential to decrease crop water stress and reduce nutrient loss below the rooting zone [29]. Conversely, biochar is able to increase porosity and permeability in fine-grained clay soils, making them more suitable for crop growth by increasing infiltration rates. Our results, combined with those of other studies, strongly support the argument that biochar addition increases the water holding capacity in coarse-grained soils, likely improving plant water availability. The saturated hydraulic conductivity (*K*) results of these laboratory and field experiments should be considered short-term, as reported changes in soil structure and hydrology are likely to evolve with time, impacting *K*. Brodowksi et al. [72] showed that biochar degrades into silt-sized particles over time likely changing the porosity and *K* of the amended soil. Other mechanisms may additional act to alter *K* on longer timescales, including increased microbial activity and increased bioturbation. Increased microbial activity and the addition of OM associated with biochar amendments has been shown to increase soil aggregation and macropore volume, thereby increasing *K* [73]. Increased bioturbation associated with biochar amendment can also increase *K* [74]. These examples provide further impetus for examining the effects of biochar particle size on soil hydraulic properties in the near- and long-term and in both laboratory/greenhouse and field settings.

Our experiments also illustrate that biochar addition to soils can add and sorb leachable DOC, and potentially add aromatic DOC to rivers [5]. Furthermore, biochar has the potential to reduce TDN in leachates, mitigating environmental impacts of agricultural N pollution. These changes in biogeochemical cycling, as well as alterations in greenhouse gas fluxes, plant productivity, and microbial activity are inherently linked to soil hydraulic properties. It is therefore crucial that future research addresses the complex interactions between biochar amendment and soil hydrology, C and N cycling, as well as how different soil types, biochars (varying feedstock, particle size, and production conditions) and amendment rates control these processes.

## Supporting Information

Table S1 **Average saturated hydraulic conductivity results for soil materials with and without biochar.** The average and standard deviation saturated hydraulic conductivity (K) for each flushing experiment (n = 3 for each soil material) in m/s. The duration of the experiment (in hours) is also provided.
(XLSX)
